# An Assessment of the Knowledge, Perception, and Willingness to Use Telepharmacy Services Among the General Public in the Kingdom of Saudi Arabia

**DOI:** 10.7759/cureus.31769

**Published:** 2022-11-22

**Authors:** Rashid H Alnajrani, Nouf R Alnajrani, Faisal S Aldakheel, Fatimah Y Alhmoud, Hajar A Al-Makenzi, Hamsa Y Zahrani, Hana A Lubbad, Hamdan N Alajami

**Affiliations:** 1 Pharmaceutical Care Services, King Saud Medical City, Ministry of Health, Riyadh, SAU; 2 College of Pharmacy, Almaarefa University, Riyadh, SAU

**Keywords:** willingness, self-perception, general public, knowledge level, telepharmacy

## Abstract

Background and objective

At the height of the coronavirus disease 2019 (COVID-19) pandemic, numerous strategies were introduced by the authorities to contain the spread of the virus, which significantly affected people's lives and impeded their mobility. As the general public was unable to leave their dwellings, many digitalized pharmacist-led services were initiated to meet the public’s needs for pharmaceutical care. The aim of this study was to ascertain the knowledge, perception, and willingness to utilize telepharmacy services and the determinants associated with these services among the general public in the Kingdom of Saudi Arabia (KSA).

Methodology

A cross-sectional survey involving participants recruited from the Saudi general public was conducted by using a validated questionnaire. We collected information regarding participants' demographics, as well as knowledge, perception, and willingness toward telepharmacy. The results were analyzed via descriptive statistics. The Mann-Whitney U Test was applied to assess the associations between knowledge, perception, willingness, and demographic variables regarding the utilization of telepharmacy services.

Results

A total of 273 Saudi citizens participated in the study; 71% (n=193) of them were aware of telepharmacy services. Many respondents showed a positive perception towards telepharmacy services and 83% (n=227) of the total participants showed their willingness to use telepharmacy services in the future. A significant association was identified between education, gender, and the knowledge of the participants regarding telepharmacy services. The demographic characteristics such as age, gender, and education, were not associated with the participants' perception regarding telepharmacy services. However, gender was significantly associated with the willingness to use telepharmacy services in the future.

Conclusions

Many participants had a fair knowledge and positive perception of telepharmacy services. More than two-thirds of the participants showed their willingness to utilize telepharmacy services in the future. However, further measures should be implemented involving strategies to increase the knowledge about telepharmacy by targeting the less educated among the Saudi population and those with limited access to technology.

## Introduction

In December 2019, cases with pneumonia-like symptoms were reported to the World Health Organization (WHO) China Country Office in Wuhan City. The WHO later revealed that cases of pneumonia of unknown etiology were caused by severe acute respiratory syndrome coronavirus 2 (SARS-CoV-2), and the condition was subsequently termed coronavirus disease 2019 (COVID-19). In January 2020, the WHO declared COVID-19 a Public Health Emergency of International Concern [1].

COVID-19 spread rapidly in the Wuhan region as well as to more than 210 countries worldwide [2]. In the Kingdom of Saudi Arabia (KSA), the first case of COVID-19 was officially announced on March 2, 2020 [3]. The KSA government enforced numerous strategies and measures to control this outbreak during the peak of COVID-19 [4]. These strategies included the requirement of wearing a face mask, self-quarantine, social distancing, and later on, the obligation to get a vaccine shot against COVID-19 [4]. Moreover, the Saudi Ministry of Health disseminated information about COVID-19 to the general public via electronic and social media in 12 different languages [4].

Telepharmacy is defined as "a method used in pharmacy practice in which a pharmacist utilizes telecommunications technology to oversee aspects of pharmacy operations or provide patient-care services" [5]. Telepharmacy has demonstrated the potential to improve the quality of pharmacy services by reducing the incidence of medicines-related problems, such as medication errors and adverse drug events [6]. In addition, telepharmacy may provide great benefits for people residing in rural areas and places that lack medical facilities and specialist services [7]. Telepharmacy is especially beneficial at times when a pharmacy professional is needed but is not available in person [7]. Telehealth services are effective in reducing strain on hospitals by reducing patient visits to hospitals [8]. Globally, telepharmacy has been utilized for the provision of consultation on chronic illnesses, and consultation for acute diseases such as COVID-19 [9].

Many community pharmacists in Jordan believed that telepharmacy facilitates patients to receive rapid medical opinions [10]. Previous studies in the United States and Jordan have reported that pharmacy students were not aware of telepharmacy but stated that it would be useful to avoid medicine-related problems and save time; they also exhibited a keen willingness to utilize the services [10,11]. A previous study conducted in KSA assessed the knowledge and attitude of pharmacists regarding telepharmacy [12]. Factors such as regulations, patients’ access to technology, and financial status may affect people’s attitudes in terms of using telepharmacy services [13]. Nonetheless, evidence regarding the Saudi public perception and knowledge about telepharmacy and their willingness to utilize this service is limited. This evidence is crucial for policymakers for developing interventions to improve the acceptability of telepharmacy among the general public. Therefore, we aimed to evaluate the perception, knowledge, and willingness to utilize the telepharmacy services, and associated factors among community-dwelling people in KSA.

## Materials and methods

We conducted a cross-sectional study between September 2022 and October 2022 among the general population in KSA. Only Saudi citizens living in KSA were eligible to participate in the study. We did not include expatriates living in KSA and those who only answered the sociodemographic questions in the questionnaire.

Anonymous data were collected from the public using an online questionnaire which was designed via Google Forms, which included queries on age, gender, education, residence, chronic disease (Yes or No), and questions regarding knowledge, willingness, and perceptions regarding telepharmacy. A 15-item self-administered questionnaire was distributed among the public living in KSA. This questionnaire was adapted from a previous study [14]. The face validity and useability of the study tool were ascertained through a pilot sample of 10 community-dwelling individuals living in KSA. Based on the piloting feedback, the study tool was also reviewed to ensure that it was easy to comprehend and complete. The questionnaire was used to collect data regarding sociodemographic information (e.g., age, gender, level of education, province of origin, and presence of chronic disease), knowledge regarding telepharmacy services, perception towards telepharmacy services, and willingness to use telepharmacy services. The perception of participants was measured by a 5-point Likert-type scale ranging from 1 (strongly disagree) to 5 (strongly agree). We divided the participants into two groups. Participants with a total score of 1-17 were deemed to have poor perceptions. Respondents with a total score of 18-35 were grouped as having good perceptions. Multiple recruitment strategies were applied to approach the public, including social media (Twitter, Facebook, LinkedIn), and via direct contact with participants visiting shopping malls and healthcare settings.

We provided a study information sheet to all participants, and also briefed them about the purpose of the study. The ethics committee approved the waiver of written informed consent. The study was initiated after obtaining ethical approval from the Institutional Review Board (reference number: H1R1-25-Sep22-02). The Strengthening the Reporting of Observational Studies in Epidemiology (STROBE) statement guidelines were followed in reporting this research study [15].

The sample size was calculated using Slovin's formula [16,17], and a minimum of 204 participants was required to obtain a 95% confidence level and a margin of error of 7% based on the total Saudi population of 34 million [18]. The respondents’ characteristics were summarized using descriptive statistics. Since the sample size was more than 50 subjects, the Kolmogorov-Smirnov test was used to determine the normality of the data. The data were not normally distributed, and hence the Mann-Whitney U test was applied to assess the associations between knowledge, perception, willingness, and demographic variables regarding the utilization of telepharmacy services. A p-value <0.05 was considered statistically significant. The statistical analysis was carried out using SPSS Statistics version 25.0 (IBM Corp., Armonk, NY).

## Results

A total of 273 participants were included in this study (response rate: 91%). The demographics of the participants are presented in Table 1. Many participants were aged between 20-30 years (n=122, 44.7%). More than half of the participants (56.8%) had a bachelor’s degree. About 61% (n=166) of the participants were from Riyadh. Of note, 233 participants (85%) did not report having a chronic disease.

**Table 1:** Demographic characteristics of the participants (n=273)

Variables	N	Percentage (%)
Age (years)		
<15	1	0.4
16–19	12	4.4
20–30	122	44.7
31–40	77	28.2
41–50	34	12.5
51–60	16	5.9
>60	11	4.0
Gender		
Male	133	48.7
Female	140	51.3
Level of education		
Middle school	11	4.0
High school	56	20.5
Bachelor's	155	56.8
Master's	27	9.9
PhD	5	1.8
Other	19	7.0
Province/State		
Jeddah	27	9.9
Riyadh	166	60.8
Dammam	17	6.2
Other	63	23.1

As mentioned above, 233 participants did not report any chronic illness and more than two-thirds of the participants (n=193, 70.7%) had heard of telepharmacy (Table 2). Many community-dwelling participants (n=165, 60.4%) had never used the telepharmacy services; however, most participants (83%) showed interest in using it.

**Table 2:** Knowledge and willingness to use telepharmacy services

Questions	N (%)	
	Yes	No
Knowledge about telepharmacy services		
Have you ever heard about telepharmacy?	193 (70.7%)	80 (29.3%)
Have you ever used telepharmacy services before?	108 (39.6%)	165 (60.4%)
Willingness to use telepharmacy services		
Are you interested in using telepharmacy services?	227 (83.2%)	46 (16.8%)

One hundred and ninety participants (70%) stated that there are numerous telepharmacy services that they can utilize in Saudi Arabia (Table 3). Many participants liked to use the telepharmacy services (n=174, 64%); however, some of the participants (n=79, 29%) felt neutral about their attitudes toward these services. Most participants agreed that telepharmacy enables people to communicate with general practitioners regardless of the time and people's locations (whenever and wherever) (n=238, 87%). Many participants endorsed the idea that the provision of telepharmacy service is crucial for saving time and energy. More than 60% of the participants (n=169) believed that telepharmacy services facilitate reducing service costs, and 164 participants showed their willingness to pay for the service. Moreover, 212 participants (77%) reported their willingness to recommend telepharmacy services to their relatives and friends.

**Table 3:** Perception of telepharmacy services

Statements	Response, n (%)				
	Strongly disagree	Disagree	Neutral	Agree	Strongly agree
There are many telepharmacy services that I can use in Saudi Arabia	5 (1.8%)	15 (5.5%)	63 (23.1%)	124 (45.4%)	66 (24.2%)
I like using telepharmacy services	2 (0.7%)	18 (6.6%)	79 (28.9%)	111 (40.7%)	63 (23.1%)
Telepharmacy service is important to be able to communicate with medical practitioners whenever and wherever	1 (0.4%)	6 (2.2%)	28 (10.3%)	93 (34.1%)	145 (53.1%)
Telepharmacy helps to save time and energy	1 (0.4%)	4 (1.5%)	32 (11.7%)	130 (47.6%)	106 (38.8%)
Telepharmacy helps to reduce service costs	4 (1.5%)	21 (7.7%)	79 (28.9%)	104 (38.1%)	65 (23.8%)
I am willing to pay for telepharmacy services	7 (2.6%)	20 (7.3%)	82 (30.0%)	115 (42.1%)	49 (17.9%)
I will recommend telepharmacy to my friends and family	1 (0.4%)	8 (2.9%)	52 (19.0%)	114 (41.8%)	98 (35.9%)

Gender (p=0.021) and educational level (p=0.026) were significantly associated with the knowledge among the public about telepharmacy services (Table 4). However, the age of the participants did not show any association with their knowledge of telepharmacy services. As for perception, we divided the cohort into respondents who had already utilized telepharmacy services (Group 1) and respondents who had never utilized any telepharmacy services (Group 2). Age, gender, and educational level were not associated with their perception regarding telepharmacy services among these two groups (Table 4). We noticed a statistically significant association between gender and the willingness of the participants to utilize the telepharmacy services (p=0.014). However, there was no significant association between age (p=0.170), educational level (p=0.462), and willingness to utilize telepharmacy services (Table 4).

**Table 4:** Association of demographic variables with knowledge, perception, and willingness to use telepharmacy Group 1: participants who had already used telepharmacy services before. Group 2: participants who had never used telepharmacy services before

	P-value
Knowledge	
Age	0.836
Gender	0.021
Level of education	0.026
Perception	
Age group - Group 1	0.119
Age group - Group 2	0.465
Gender - Group 1	0.469
Gender - Group 2	0.530
Level of education - Group 1	0.322
Level of education - Group 2	0.324
Willingness	
Age	0.170
Gender	0.014
Level of education	0.462

## Discussion

Telepharmacy services are crucial for increasing patients' access to pharmaceutical care and reducing medicines-related problems [19]. Moreover, increased patient access to telepharmacy services may improve patient outcomes and prevent hospitalizations [20]. It is prudent to assess the knowledge, willingness, and perception of the community-dwelling people towards telepharmacy services for developing an effective educational strategy, thereby facilitating the access of patients to pharmaceutical care via telepharmacy services.

More than two-thirds of our study participants had heard about telepharmacy services. Many respondents demonstrated a good perception of the telepharmacy services that had been provided to them in the past. Although over half of the participants had not utilized telepharmacy services, more than 80% of the participants showed their willingness to utilize telepharmacy services in the future. The level of education and gender were associated with the knowledge of participants regarding telepharmacy services. A previous study has also reported a significant association between education and knowledge of participants about telepharmacy services [14], which is consistent with our study findings. We did not find an association between demographic characteristics and the perception of participants towards telepharmacy services, which is concordant with the findings of a study conducted in Indonesia [14]. That study did not report any association between demographic characteristics and the willingness of Indonesian people towards telepharmacy services [14]. However, we noticed an association between the willingness of the general public towards utilizing telepharmacy services and their gender.

The study findings showed that about 71% of the respondents had already heard about telepharmacy. This percentage was relatively higher compared to that in studies performed in India (18.9%) [21] and Indonesia (51%) [14]. In addition, many of our study participants who had never utilized telepharmacy services in the past showed their willingness to utilize them in the future. This is consistent with the findings of a previous report that the usage of telemedicine has been increasing since the COVID-19 outbreak [22]. This is supported by the fact that although 85% of the study respondents were healthy (e.g., without any chronic illness), they had already heard of telepharmacy before participating in the study. There is a possibility that the strict COVID-19 restrictions in KSA prompted people to use online services (e.g., telepharmacy) for getting access to pharmaceutical care.

We noticed that most of our participants reported a positive perception of telepharmacy services. The participants believed that telepharmacy could benefit with regard to cutting time, cost, and energy. Furthermore, participants agreed that telepharmacy services could turn out to be more convenient as it is provided online. This finding is in line with that of an earlier report that the consumers who utilized telemedicine healthcare were extremely satisfied with the provision of services rendered [23]. Telemedicine has been recognized to improve the quality of care for the diabetic population in KSA via effective monitoring and maintaining their glucose levels [24].

We identified that there was a significant association between gender and the participant's knowledge regarding telepharmacy services in KSA. This is probably due to the differences in the level of experience with online technology between males and females. Females tend to use technology more frequently as they spend most of their time at home, and hence may have more knowledge about the recently introduced services. According to the World Bank, the labor force participation rate among females (31%) is significantly lower than among males (80%) in KSA [25].

The respondents with higher educational levels (e.g., bachelor’s degrees) in our study had apparently more knowledge about telepharmacy services. This could be attributed to the fact that people with higher educational levels were exposed to more technology. This finding is in line with an Egyptian study that reported that highly qualified individuals have more awareness of telemedicine services [26]. We also noticed that age was not significantly associated with the knowledge of participants regarding telepharmacy services, which is probably due to the fact that in the current digital age, all residents have access to the internet regardless of their age. In this study, there was no association between the demographic characteristics (e.g., age, gender, and education) and participants' perception of telepharmacy services. This could be attributed to the knowledge of the public regarding the importance of sustaining good health. In KSA, there is an adequate opportunity via social media and online counseling for all residents regardless of their age group, gender, and level of education.

Our study results suggest that there is a gap between knowledge of telepharmacy among both genders and their level of education. There is a need to implement health promotion activities that target all residents regardless of their educational qualifications and whether they stay at home or work outside. Health promotion activities could also be conducted in educational and healthcare institutions to ensure that all residents are aware of the new regulations about telepharmacy services.

This is the first nationwide study of its kind conducted among the general public in KSA that assessed the knowledge, willingness, and perception of community-dwelling people towards telepharmacy services as well as the factors associated with them. Evidence indicates that previous studies primarily focused on pharmacists [27-29] while findings related to community-dwelling people are limited. However, this study also has certain limitations. Many respondents were university graduates and without any chronic illnesses, and hence our findings are probably not representative of the general population in KSA. We recommend that caution be exercised in interpreting and extrapolating our findings. Further research involving qualitative interviews should be conducted to gain deeper insights and comprehension regarding the utilization of telepharmacy services by the public.

## Conclusions

Many participants in our study demonstrated a fair knowledge and positive perception towards telepharmacy services. More than two-thirds of the participants showed their willingness to utilize telepharmacy services in the future. Interventions to increase awareness of telepharmacy in KSA need to target the less educated among its population and those with limited access to technology, which may improve the acceptability of telepharmacy services among the general public.
